# Molecular Evidence of Lateral Gene Transfer in *rpoB* Gene of *Mycobacterium yongonense* Strains via Multilocus Sequence Analysis

**DOI:** 10.1371/journal.pone.0051846

**Published:** 2013-01-31

**Authors:** Byoung-Jun Kim, Seok-Hyun Hong, Yoon-Hoh Kook, Bum-Joon Kim

**Affiliations:** Department of Microbiology and Immunology, Cancer Research Institute, Institute of Endemic Diseases, Seoul National University Medical Research Center (SNUMRC), Seoul National University College of Medicine, Seoul, Republic of Korea; Hopital Raymond Poincare – Universite Versailles St. Quentin, France

## Abstract

Recently, a novel species, *Mycobacterium yongonense* (DSM 45126^T^), was introduced and while it is phylogenetically related to *Mycobacterium intracellulare*, it has a distinct RNA polymerase β-subunit gene (*rpoB*) sequence that is identical to that of *Mycobacterium parascrofulaceum*, which is a distantly related scotochromogen, which suggests the acquisition of the *rpoB* gene via a potential lateral gene transfer (LGT) event. The aims of this study are to prove the presence of the LGT event in the *rpoB* gene of the *M. yongonense* strains via multilocus sequence analysis (MLSA). In order to determine the potential of an LGT event in the *rpoB* gene of the *M. yongonense*, the MLSA based on full *rpoB* sequences (3447 or 3450 bp) and on partial sequences of five other targets [16S rRNA (1383 or 1395 bp), *hsp65* (603 bp), *dnaJ* (192 bp), *recA* (1053 bp), and *sodA* (501 bp)] were conducted. Incongruences between the phylogenetic analysis of the full *rpoB* and the five other genes in a total of three *M. yongonense* strains [two clinical strains (MOTT-12 and MOTT-27) and one type strain (DSM 45126^T^)] were observed, suggesting that *rpoB* gene of three *M. yongonense* strains may have been acquired very recently via an LGT event from *M. parascrofulaceum*, which is a distantly related scotochromogen.

## Introduction

From a clinical and epidemiological perspective, the members of the *Mycobacterium avium* complex (MAC) are the most important nontuberculous mycobacteria (NTM). Traditionally, MAC includes two species, *M. avium* and *M. intracellulare* [1], [2], [3]; in Korea, the prevalence of *M. intracellulare* infections is higher than that of *M. avium* [4]. Recently, it was reported that *M. intracellulare* related strains from Korean patients showed more genetic diversity; the strains can be divided into a total of five distinct groups using the sequence analysis of *hsp65*, internal transcribed spacer and 16S rRNA genes [5].

Generally, the informative genes associated with the central dogma of bacteria, such as the 16S rRNA gene or the RNA polymerase gene (*rpoB*), have been reported to be recalcitrant to lateral gene transfer (LGT) events. However, the LGT events of informative genes within the genus *Mycobacterium* have been disclosed in two recent reports. One report described the potential LGT event of the *rpoB* gene between three groups of strains belonging to *Mycobacterium abscessus* (*M. abscessus sensu stricto, Mycobacterium massiliense* and *Mycobacterium bolletii*) [6]; the other report described the potential LGT event of the 16S rRNA gene between *Mycobacterium franklinii* and *Mycobacterium chelonae* [7]. Moreover, a novel species, *M. yongonense*, which is phylogenetically related to *M. intracellulare*, was introduced from studies of a Korean patient with pulmonary symptoms. Notably, *M. yongonense* proved to have a distinct RNA polymerase gene (*rpoB*) sequence identical to that of *M. parascrofulaceum*, which is a distantly related scotochromogen, suggesting that the *rpoB* gene was acquired via a potential LGT event [8].

The aims of the current study are two-fold: the first is to discover the epidemiologic features of *M. yongonense* from an infection cohort previously identified as *M. intracellulare* and the second is to prove the presence of the LGT event in the *rpoB* gene of the *M. yongonense* strains via multilocus sequence analysis (MLSA). In order to determine the potential of an LGT event in the *rpoB* gene of *M. yongonense*, the MLSA based on full *rpoB* sequences (3447 or 3450 bp) and partial sequences of the other five targets [16S rRNA (1383 or 1395 bp), *hsp65* (603 bp), *dnaJ* (192 bp), *recA* (1053 bp), and *sodA* (501 bp)] were applied to a total of seven mycobacteria strains: three *M. yongonense* (MOTT-12, MOTT-27 and DSM 45126^T^), two *M. intracellulare* strains (MOTT-02 and ATCC 13950^T^), and two *M. parascrofulaceum* strains (MOTT-01 and ATCC BAA-614^T^).

## Methods

### Mycobacterial isolates

Seven mycobacteria strains, including three reference strains (*M. intracellulare* ATCC 13950^T^, *M. parascrofulaceum* ATCC BAA-614^T^ and *M. yongonense* DSM 45126^T^) and four clinical isolates (MOTT-01, MOTT-02, MOTT-12, and MOTT-27) were analyzed using the MLSA (Table S1). Of the four clinical isolates, one (MOTT-01) was identified as *M. parascrofulaceum*, one (MOTT-02) as *M. intracellulare* and two (MOTT-12 and MOTT-27) as *M. yongonense*, using a combination of the *hsp65* and *rpoB* sequence based analyses. The experiment was based entirely on the extracted genomic DNA from the isolates, so the research was undertaken without informed consent and a waiver of informed consent was obtained from the Institutional Review Board (IRB) of Seoul National University Hospital. This work was approved by the IRB of Seoul National University Hospital (C-1204-003-403).

### Biochemical tests

In order to identify and differentiate the two *M. yongonense* clinical isolates (MOTT-12 and MOTT-27), their biochemical test profiles were compared with those of three type reference strains: *M. intracellulare* ATCC 13950^T^, *M. yongonense* DSM 45126^T^ and *M. parascrofulaceum* ATCC BAA-614^T^. The colony morphology, pigmentation in the dark, photo-induction and growth at different temperatures (25°C, 37°C and 45°C) were tested on 7H10 agar plates with OADC over a six-week incubation period. The acid-alcohol-fastness was examined via Ziehl-Neelsen and auramine O staining. The biochemical characteristics of niacin accumulation, nitrate reductase, arylsulfatase on Days 3 and 14, and the heat-stable catalase (pH 7, 68°C), tellurite reductase, Tween 80 hydrolysis, urease and pyrazinamidase were tested [10]. The inhibition tests including the tolerance to thiophene-2-carboxylic acid hydrazide (TCH), p-nitrobenzoate (PNB), 5% sodium chloride, ethambutol (EMB), and picric acid were performed; and the ability to grow on MacConkey agar without crystal violet was also examined.

### Sequence analysis of full *rpoB* gene and five other genes [16S rRNA (1383 or 1395 bp), *hsp65* (603 bp), *dnaJ* (192 bp), *recA* (1053 bp), and *sodA* (501 bp)]

In order to verify the LGT of the *rpoB* gene between the *M. parascrofulaceum* and the three *M. yongonense* strains (MOTT-12, MOTT-27, and DSM 45126^T^), the full *rpoB* gene sequences (3447 or 3450 bp) and sequences from five other targets [16S rRNA (1383 or 1395 bp), *hsp65* (603 bp), *dnaJ* (192 bp), *recA* (1053 bp), and *sodA* (501 bp)] of the four clinical and three reference stains were analyzed. The bead beater-phenol extraction method was used to extract the chromosomal DNA of these strains, as previously reported [9]; the extracted DNA samples were then used as templates for the polymerase chain reaction (PCR) amplifications of the six independent sequence targets [*rpoB* (partial and complete), 16S rRNA, *hsp65*, *dnaJ*, *recA*, and *sodA*]. The PCR amplifications were bi-directionally sequenced using the same primers as those used in the PCR. The PCR amplification and sequence analysis of the *rpoB* (partial and complete), 16S rRNA, *hsp65*, *dnaJ*, *recA*, and *sodA* genes were performed as described previously [5], [9], [11], [12], [13], [14]. A total of six primer sets were used for the amplification of the full *rpoB* gene (3447 or 3450 bp) sequence. The locations and sequences of the primers for the *rpoB* amplification are shown in Figure S1 and Table S2, respectively. These primer sets were designed using the whole genome sequence database of *M. intracellulare* ATCC 13950^T^ (GenBank no. ZP_05227774) and *M. avium* 104 (GenBank no. NC_008595). The sequences of the primers for the amplification and sequencing of the *rpoB* (partial and complete), 16S rRNA, *hsp65*, *dnaJ*, *recA*, and *sodA* genes are also shown in Table S2. For the phylogenetic analysis of the *rpoB* (partial and complete), 16S rRNA, *hsp65*, *dnaJ*, *recA*, and *sodA* genes, the nucleotide sequence similarities of each gene were determined using the MegAlign package (DNASTAR) software. The phylogenetic trees were constructed from the full sequences of the *rpoB gene* (3447 or 3450 bp), the partial sequences of four genes [*hsp65* (603-bp), *dnaJ* (192 bp), *recA* (1053 bp), and *sodA* (501 bp)] and 16S rRNA (1383 or 1395 bp) sequences using the neighbor-joining method [15] in the MEGA 4 software; the bootstrap values were calculated from 1,000 replications [16].

### Nucleotide accession numbers

The sequences of the seven target genes [*hsp65* gene (603 bp), 16S rRNA (1383 or 1395 bp), the *rpoB* gene (306 bp), the full *rpoB* gene (3447 or 3450 bp), *dnaJ* (192 bp), *recA* (1053 bp), and *sodA* (501 bp)] from a total of seven strains, including three reference (*M. intracellulare* ATCC 13950^T^, *M. parascrofulaceum* ATCC BAA-614^T^, and *M. yongonense* DSM 45126^T^) and four clinical (MOTT-01, MOTT-02, MOTT-12, and MOTT-27) strains were deposited in the GenBank database; the GenBank numbers are presented in Table S1. Among these, the *hsp65* (FJ849777) gene sequences of the MOTT-27 strain were retrieved from a previous report by Park *et* *al*. [5] (Table S1).

## Results and Discussion

### Characterization of the phenotypic traits of the two *M. yongonense* clinical strains (MOTT-12 and MOTT-27) based on conventional biochemical tests

The conventional taxonomic approaches based on biochemical traits demonstrated that all strains shared similar growth patterns. Pigmentation is known to be the most pronounced difference between *M. intracellulare* and *M. parascrofulaceum*; the former is a nonphotochromogen; however, the latter is a scotochromogen [17]. The two *M. yongonense* clinical strains in the current study (MOTT-12 and MOTT-27) proved to be nonchromogens, suggesting that they are phenotypically closer to *M. intracellulare*, rather than *M. parascrofulaceum* as described previously [8]. However, the differences in some biochemical traits such as nitrate reductase, arylsulfatase and tellurite reductase were found between *M. yongonense* DSM 45126^T^ and the two clinical strains (MOTT-12 and MOTT-27) (see Table S3).

### Molecular taxonomy of the three *M. yongonense* isolates (MOTT-12, MOTT-27, and DSM 45126^T^) via phylogenetic analysis based on full *rpoB* sequences

**Figure 1:**
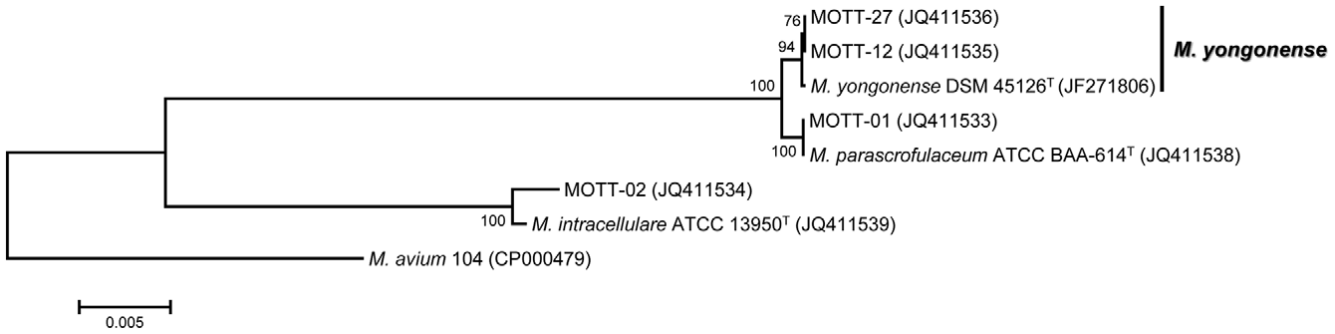
Phylogenetic relationships based on the full *rpoB* gene (3447 or 3450 bp) sequences. This tree was constructed using the neighbor-joining method. The bootstrap values were calculated from 1,000 replications; bootstrap values of <50% are not shown.

In order to prove the hypothesis that there may have been an LGT event for the *rpoB* gene between *M. yongonense* and *M. parascrofulaceum*, the full *rpoB* sequences of seven strains, including the three *M. yongonense* strains [two clinical strains (MOTT-12 and MOTT-27) and one type strain (DSM 45126^T^)], were analyzed. The full *rpoB* gene sequence proved useful for the delineation of the bacterial species [18]. A *rpoB* gene sequence similarity of <97.0% is reported to be significantly correlated with a DNA-DNA hybridization (DDH) value of <70%, which is the universal cut-off value for the delineation of a bacterial species [18]. All full length *rpoB* sequences obtained in the current study were verified to be encoded in the proper deduced RpoB amino acids in the *in silico* translation. The phylogenetic analysis based on the full *rpoB* sequences (3450 bp) demonstrated that the three *M. yongonense* isolates (MOTT-12, MOTT-27, and DSM 45126^T^) formed a tight cluster with the *M. parascrofulaceum* strains (ATCC BAA-614^T^ and MOTT-01) rather than with the *M. intracellulare* strains (ATCC 13950^T^ and MOTT-02). Also their phylogenetic relationship was supported by a high bootstrap value (100.0; Figure 1). The sequence similarity value of the full *rpoB* sequences between the three *M. yongonense* strains and two *M. parascrofulaceum* strains ranged from 99.7% to 99.8%, which presented eight to nine bp mismatches among 3450 bp. However, the sequence similarity values between the three *M. yongonense* strains and two *M. intracellulare* strains ranged from 94.7% to 94.9%, which presented 181 to 196 bp mismatches from 3450 bp (Table 1). The high similarity value observed between the *M. yongonense* and *M. parascrofulaceum* strains indicates that these two different species share almost identical *rpoB* sequences. Furthermore, the similarity values observed between the *M. yongonense* and *M. intracellulare* strains are lower than that of the cut-off value (97.0%) for the delineation of bacterial species [18] (Table 1).

**Table 1:** Full *rpoB* gene sequence (3447 and 3450 bp; right upper side) and concatenated sequence [16S rRNA (1383 or 1395 bp) + *hsp65* (603 bp) + *sodA* (501 bp) + *recA* (1053 bp) + *dnaJ* (192 bp); left down side] similarities between seven mycobacterial strains.

Strains	Sequence similarity (%)							
	MOTT-01	MOTT-02	MOTT-12	MOTT-27	Mint a	Myon b	Mpara c	Mav d
MOTT-01		94.6	99.8	99.8	94.8	99.7	100.0	94.6
MOTT-02	94.3		94.7	94.7	99.7	94.7	94.7	95.6
MOTT-12	94.2	99.7		100.0	94.8	100.0	99.8	94.5
MOTT-27	94.2	99.7	100.0		94.8	100.0	99.8	94.5
Mint	94.4	99.9	99.7	99.7		94.9	94.8	95.7
Myon	94.1	99.5	99.6	99.6	99.5		99.7	94.5
Mpara	100.0	94.3	94.2	94.2	94.4	94.1		94.6
Mav	94.2	96.8	96.7	96.7	96.9	96.8	94.2	

a Mint, *M. intracellulare* ATCC 13950 ^T^.

b Myon, *M. yongonense* DSM 45126^T^.

c Mpara, *M. parascrofulaceum* ATCC BAA-614 ^T^.

d Mav, *M. avium* 104.

### Phylogenetic analysis based on the 16S rRNA and *hsp65* gene

In order to verify the above hypothesis, a phylogenetic analysis of the three *M. yongonense* strains was performed using two other genes (16S rRNA and *hsp65* genes), which have been used widely for mycobacteria taxonomies and diagnostics [13], [19], [20], [21]. Despite some problems in the bacteria taxonomy, the 16S rRNA gene sequence-based comparisons have been and remain invaluable in describing the prokaryotic diversity; they are indispensable in the delineation of bacterial species [22]. The phylogenetic analysis based on the 16S rRNA sequence (1383 or 1395 bp) indicated that the three *M. yongonense* strains belonged to the *M. intracellulare* group, exhibiting a sequence similarity ranging from 99.8% to 100% with two other *M. intracellulare* strains (ATCC 13950^T^ and MOTT-02; data not shown). The three *M. yongonense* strains exhibited a relatively low level of similarity value (96.8%) with the *M. parascrofulaceum* strains, which was lower than the universally accepted cut-off value for the 16S rRNA gene (97.0%) for bacteria species delineation (data not shown) [23]. This strongly suggests that the three *M. yongonense* strains are phylogenetically related to *M. intracellulare*.

The *hsp65* gene sequence based methods have been the most widely used methods for mycobacteria taxonomies as alternatives to the 16S rRNA based methods [9], [13]. The three *M. yongonense* strains exhibited some minor variations (99.3% similarity value with four base pair mismatches of the 603 bp *hsp65* sequences) compared with the other two *M. intracellulare* strains (ATCC 13950^T^ and MOTT-02). The phylogenetic analysis based on the *hsp65* gene sequence (603 bp) indicated that the three *M. yongonense* strains belonged to the *M. intracellulare* group, rather than to the *M. parascrofulaceum* group, which indicates a low level of sequence similarity value of 94.9% with the two *M. parascrofulaceum* strains (ATCC BAA-614^T^ and MOTT-01; data not shown). This also strongly supports their phylogenetic location in *M. intracellulare*.

### Phylogenetic analysis based on *dnaJ* (192 bp), *recA* (1053 bp), and *sodA* (501 bp)

In order to strengthen the above hypothesis, further phylogenetic analyses were performed based on three other genes [*dnaJ* (192 bp), *recA* (1053 bp), and *sodA* (501 bp)], which have been successfully applied in mycobacteria taxonomy [11], [14].

The phylogenetic analyses based on the *dnaJ* gene sequence (192 bp) indicated that the three *M. yongonense* strains belong to the *M. intracellulare* groups and exhibited sequence similarity values of 99.5% with the other two *M. intracellulare* strains (ATCC 13950^T^, and MOTT-02). It was also clear that the three *M. yongonense* strains do not belong to the *M. parascrofulaceum* group (similarity value of 93.2%; data not shown). The phylogenetic analyses based on the *recA* gene sequence (1053 bp) also indicated that the three *M. yongonense* strains belonged to the *M. intracellulare* groups, which exhibited sequence similarity values of 99.4% to 99.6% with the other two *M. intracellulare* strains (ATCC 13950^T^ and MOTT-02), rather than with the *M. parascrofulaceum* group (similarity value of 95.4%; data not shown). The phylogenetic analyses based on the *sodA* gene sequence (501 bp) also indicated that the three *M. yongonense* strains belonged to the *M. intracellulare* groups, which exhibited sequence similarity values of 99.4% to 99.6% with the other two *M. intracellulare* strains, rather than with the *M. parascrofulaceum* group (similarity value of 78.8% to 79.0%; data not shown). Thus, all phylogenetic analyses based on the other three genes [*dnaJ* (192 bp), *recA* (1053 bp), and *sodA* (501 bp)] also confirmed that the three *M. yongonense* strains with the distinct *rpoB* gene are more closely related to *M. intracellulare* than to *M. parascrofulaceum* as shown in the phylogenetic analyses based on the 16S rRNA and *hsp65* genes.

### Phylogenetic analysis based on concatenated sequences of the five MLSA genes and the full *rpoB* gene

**Figure 2:**
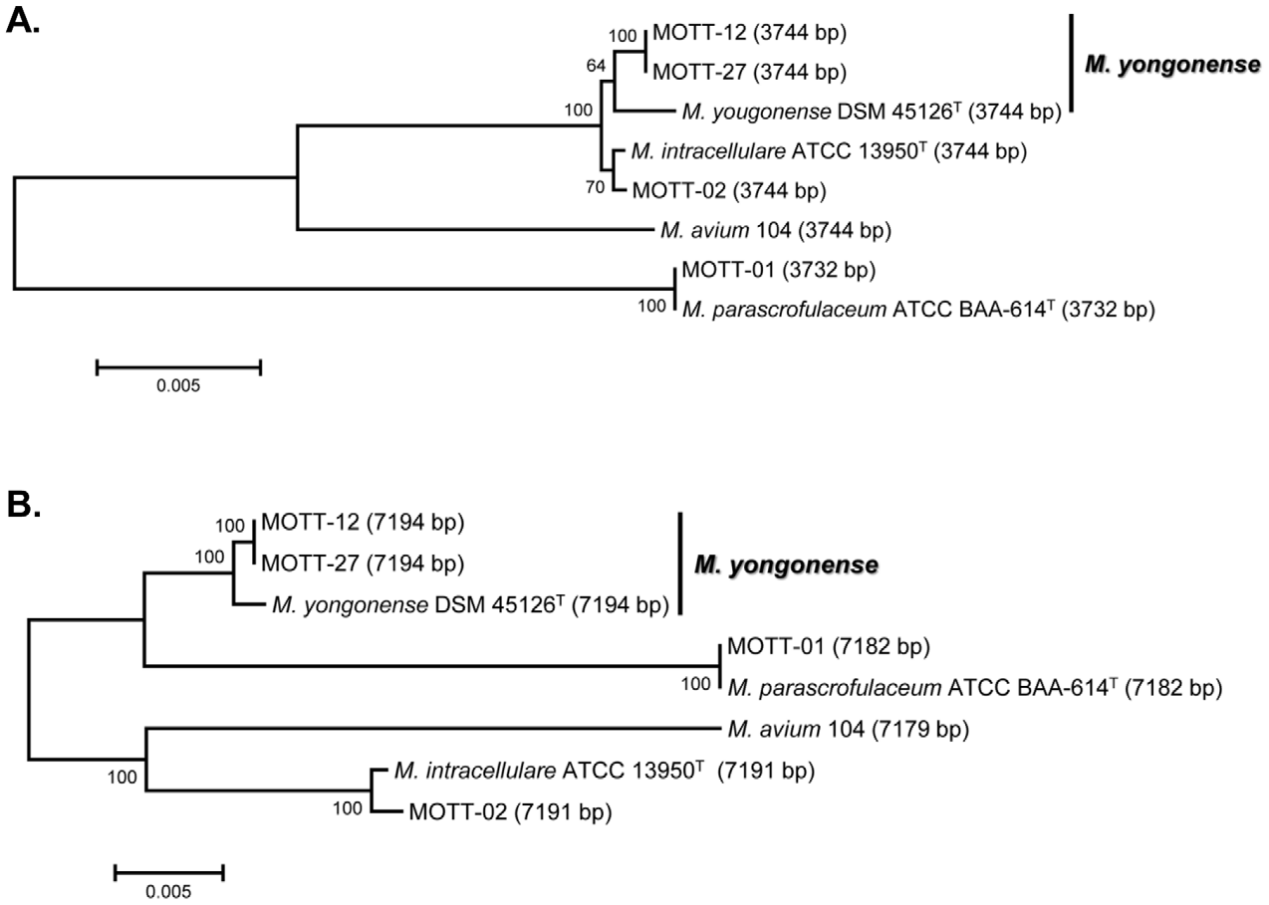
Phylogenetic relationships based on concatenated sequences of (A) the five MLSA genes (16S rRNA, *hsp65*, *dnaJ*, *recA* and *sodA*) (3732 or 3744 bp) and (B) with the addition of the full *rpoB* sequence to the concatenated sequences of the five MLSA genes (7182–7194 bp) (B). These trees were constructed using the neighbor-joining method. The bootstrap values were calculated from 1,000 replications; bootstrap values of <50% are not shown.

Figure 2A shows the tree for the seven strains obtained by concatenating the sequences of the five MLSA genes (16S rRNA, *hsp65*, *dnaJ*, *recA,* and *sodA*) (3732 or 3744 bp). The tree displays two clearly separated clusters: one for the *M. intracellulare* related strains (three *M. yongonense* and two *M. intracellulare*) and the other for the two *M. parascrofulaceum* strains. A high level of bootstrap values (100%) was observed for the groupings. The three *M. yongonense* and two *M. intracellulare* strains formed two different branches in one cluster, which indicates their phylogenetic separation. The bootstrap values of both branches were 64% (*M. yongonense*) and 70% (*M. intracellulare*). Although a complete sequence similarity between the two clinical strains (MOTT-12 and MOTT-27) was found, some variations (99.6% of 3744-bp MLSA sequences) between the clinical strains and the type strain (DSM 45126^T^) were found, which indicates that the two clinical strains may be variants of *M. yongonense* DSM 45126^T^ (Table 1).

The effect of adding the *rpoB* sequence to the concatenated sequences of the five MLSA genes (7182–7194 bp) was also studied. The topology of the obtained tree (Figure 2B) was radically different from only that constructed from the MLSA gene sequences (Figure 2A). The branch of the *M. yongonense* strains forming the same cluster with that of the *M. intracellulare* strains in the MLSA tree were transferred into a cluster belonging to the *M. parascrofulaceum* strains in the MLSA + *rpoB* tree, which was strongly supported with a high level of bootstrap values (100%). The discrepancy observed between the topology structures of both trees suggests the potential LGT event of the *rpoB* gene from the *M. parascrofulaceum* strain into the *M. yongonense* strain.

From a clinical perspective, these results emphasize the importance of the MLSA for mycobacteria identification. Currently, the *rpoB* gene has been used widely as a target gene for bacterial identification, particularly for mycobacteria identification [9], [24], [25]. However, the data in this study implies that some strains of *M. yongonense* could be misidentified as *M. parascrofulaceum* when only a single *rpoB* gene is used in the identification or as *M. intracellulare* with use of chronometers other than the *rpoB* gene.

In conclusion, collective consideration of the molecular taxonomic data based on the full *rpoB* and five other genes, which have been used widely for mycobacterial identification has led to the conclusion that the three *M. yongonense* strains with the signature *rpoB* gene have potentially acquired their *rpoB* gene via a very recent LGT event from *M. parascrofulaceum*. However, the details of the LGT events between *M. parascrofulaceum* and *M. yongonense* strains must be further elucidated in a future study. Furthermore, the data presented here also suggests that the *rpoB* gene analysis alone may have potential for misidentification in mycobacteria diagnostics. Thus, an approach using multilocus genes should be conducted for mycobacteria identification.

## Supporting Information

Figure S1 **Locations of primers used for amplification of the full** ***rpoB*** (**3450 bp**) **gene sequence in this study.**
(DOCX)

Table S1 **Mycobacteria strains used in this study.**
(DOC)

Table S2 **The primer sets used for amplification of the full** ***rpoB*** **, partial** ***rpoB*** **,** ***hsp65*** **, 16S rRNA,** ***dnaJ*** **,** ***recA*** **, and** ***sodA*** **in this study.**
(DOC)

Table S3 **Comparison of phenetic and biochemical characteristics between** ***M. yongonense*** **DSM 45126^T^, MOTT-12, MOTT-27,** ***M. parascrofulaceum*** **ATCC BAA-614^T^ and** ***M. intracellulare*** **ATCC 13950^T^.** All strains showed negative results in niacin accumulation test, and positive results in heat stable catalase test.
(DOC)
