# Supplementary figures and images for: Molecular Evidence of Lateral Gene Transfer in rpoB Gene of Mycobacterium yongonense Strains via Multilocus Sequence Analysis
Source: PLoS One. 2013 Jan 31;8(1):e51846. doi: 10.1371/journal.pone.0051846 (PMC3561371; doi:10.1371/journal.pone.0051846)

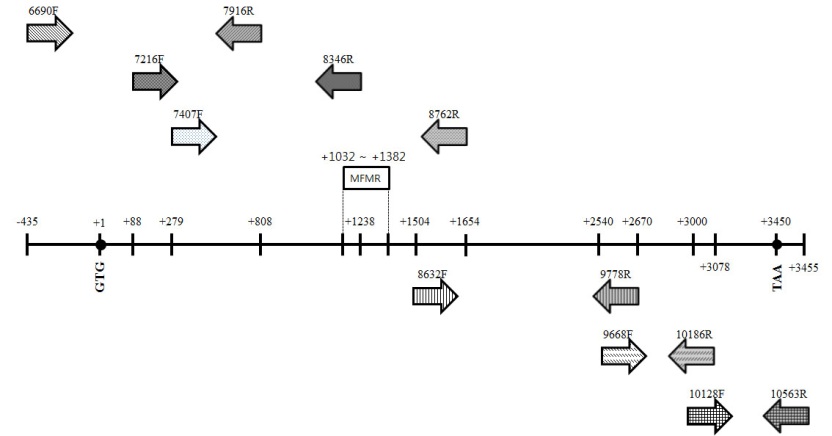

Supplement: Figure S1 — Locations of primers used for amplification of the full rpoB (3450 bp) gene sequence in this study. (DOCX) [file pone.0051846.s001.docx]
