# Supplementary material for: Molecular Evidence of Lateral Gene Transfer in rpoB Gene of Mycobacterium yongonense Strains via Multilocus Sequence Analysis
Source: PLoS One. 2013 Jan 31;8(1):e51846. doi: 10.1371/journal.pone.0051846 (PMC3561371; doi:10.1371/journal.pone.0051846)
Supplement: Table S1 — Mycobacteria strains used in this study. (DOC) [file pone.0051846.s002.doc]

| **Strains** | **Source** | **Sp. or Str. *^a^*** | ***rpoB*** | ***rpoB*** | ***hsp65*** | **16S rRNA** | ***dnaJ*** | ***recA*** | ***sodA*** |
| --- | --- | --- | --- | --- | --- | --- | --- | --- | --- |
| MOTT – 01 | AMC *^b^* | *M. parascrofulaceum* | JF271835 | JQ411533 | JF271814 | JF271822 | JQ411540 | JQ411519 | JQ411512 |
| MOTT – 02 | AMC | INT2 | JF271830 | JQ411534 | JF271808 | JF271816 | JQ411541 | JQ411520 | JQ411513 |
| MOTT – 12 | AMC | INT5 | JF271833 | JQ411535 | JF271812 | JF271820 | JQ411542 | JQ411521 | JQ411514 |
| MOTT – 27 | AMC | INT5 | JF271832 | JQ411536 | FJ849777 *^d^* | JF271819 | JQ411543 | JQ411522 | JQ411515 |
| *M. yongonense* | DSM 45126 | *M. yongonense* | JF271806 | JF271806 | JF271809 | JF271817 | JQ937025 | JQ937024 | JQ937023 |
| *M. intracellulare* | ATCC *^c^* 13950^T^ | INT2 | AF057472 | JQ411539 | JF271810 | JF271818 | JQ411546 | JQ411525 | JQ411518 |
| *M. parascrofulaceum* | ATCC BAA-614^T^ | *M. parascrofulaceum* | JF271829 | JQ411538 | JF271807 | JF271815 | JQ411545 | JQ411524 | JQ411517 |

*^a^* Separation into species or strain level were performed by via combination of *hsp65,* ITS-1 and 16s rRNA sequence based analyses .

*^b^* AMC, Asan medical center

*^c^* ATCC, American type culture collection

*^d^* Retrieved sequences from a previous report [5].
