# Supplementary material for: Molecular Evidence of Lateral Gene Transfer in rpoB Gene of Mycobacterium yongonense Strains via Multilocus Sequence Analysis
Source: PLoS One. 2013 Jan 31;8(1):e51846. doi: 10.1371/journal.pone.0051846 (PMC3561371; doi:10.1371/journal.pone.0051846)
Supplement: Table S2 — The primer sets used for amplification of the full rpoB , partial rpoB , hsp65 , 16S rRNA, dnaJ , recA , and sodA in this study. (DOC) [file pone.0051846.s003.doc]

| Genes  (Aligned sequence length) | Primer | Primer sequences (5’ to 3’) | Amplicon | Reference |
| --- | --- | --- | --- | --- |
| *rpoB*  (3447 or 3450 bp) | 6690F | CTT CGT GTA ACT CGG CGG CCC | 1247 bp | − |
|  | 7916R | AGA ACA GGT TCT CCA GCA GGG |  |  |
|  | 7216 F | TTC GAG TGG TTG ATC GGC TCG | 1151 bp |  |
|  | 8346 R | AAC TGG GAG AGC TGG CTG GTG |  |  |
|  | 7407 F | CAA GGA CAT GAC GTA CGC GGC | 1376 bp |  |
|  | 8762 R | AGT CCA CCT CGG ACG ACG GCA |  |  |
|  | 8632 F | ATC CAC TAC CTG ACC GCC GAC | 1167 bp |  |
|  | 9778 R | GAT GAT GTC CAC CGG CGT GCC |  |  |
|  | 9668 F | TGG CCC AGA AGC GCA AGA TCT | 539 bp |  |
|  | 10186 R | CTG CTG GGT GAT CAT CGA GTA |  |  |
|  | 10128 F | CAT CAT GAA GCT GCA CCA CCT | 456 bp |  |
|  | 10563 R | AGT GAT TAA GCC AGG TCC TCA |  |  |
| *rpoB* (306 bp) | MF | CGA CCA CTT CGG CAA CCG | 351 bp | [5, 9] |
|  | MR | TCG ATC GGG CAC ATC CGG |  |  |
| *hsp65* (603 bp) | HspF3 | ATC GCC AAG GAG ATC GAG CT | 644 bp | [5, 9] |
|  | HspR4 | AAG GTG CCG CGG ATC TTG TT |  | [5, 9] |
| 16S rRNA  (1383 or 1395 bp) | 285 | GAG AGT TTG ATC CTG GCT CA | 1525 bp | [5, 9] |
|  | 261 | AAG GAG GTG ATC CAG CCG CA |  |  |
| *dnaJ* (192 bp) | F | GGG TGA CGC G(G/A)C ATG GCC CA | 236 bp | [11, 14] |
|  | R | CGG GTT TCG TCG TAC TCC TT |  |  |
| *sodA* (501 bp) | SodlgF | GAA GGA ATC TCG TGG CTG AAT AC | 547 bp | [11, 14] |
|  | SodlgR | AGT CGG CCT TGA CGT TCT TGT AC |  |  |
| *recA* (1053 bp) | RecF1 | GGT GTT CGN CTA NTG TGG TG | 703 bp | [11] |
|  | RecR1 | AGC TGG TTG ATG AAG ATY GC |  |  |
|  | RecG1 | CTS GAR ATC GCC GAC ATG CTG | 596 bp |  |
|  | RecR2 | TTG ATC TTC TTC TCG ATC TC |  |  |
|  | Rec3288F | CAA GCA GGC CGA GTT CGA CAT C | 319 bp |  |
|  | Rec3575R | AGG ATC CTG CGC CTG CTC |  |  |
