# Supplementary material for: Molecular Evidence of Lateral Gene Transfer in rpoB Gene of Mycobacterium yongonense Strains via Multilocus Sequence Analysis
Source: PLoS One. 2013 Jan 31;8(1):e51846. doi: 10.1371/journal.pone.0051846 (PMC3561371; doi:10.1371/journal.pone.0051846)
Supplement: Table S3 — Comparison of phenetic and biochemical characteristics between M. yongonense DSM 45126T, MOTT-12, MOTT-27, M. parascrofulaceum ATCC BAA-614T and M. intracellulare ATCC 13950T. All strains showed negative results in niacin accumulation test, and positive results in heat stable catalase test. (DOC) [file pone.0051846.s004.doc]

| **Characteristics** | ***M. yongonense*** | **MOTT-12** | **MOTT-27** | ***M. parascrofulaceum*** | ***M. intracellulare*** |
| --- | --- | --- | --- | --- | --- |
| Growth at: |  |  |  |  |  |
| 25 ℃ | + | + | + | – | + |
| 37 ℃ | ++ | ++ | ++ | +++ | ++ |
| 45 ℃ | – | – | – | – | – |
| Growth detectable after: |  |  |  |  |  |
| < 7 days | – | – | – | – | – |
| > 7 days | ++ | +++ | +++ | +++ | ++ |
| Morphology ^†^ | SWY | SWY | IWY | SWY | IWY |
| Pigmentation ^‡^ | N | N | N | S | N |
| Nitrate reductase | – | ± | ± | – | – |
| Arylsulfatase |  |  |  |  |  |
| 3 days | ± | – | – | – | ± |
| 14 days | + | – | – | – | + |
| Tellurite reductase | + | – | – | – | + |
| Tween hydrolysis |  |  |  |  |  |
| < 5 days | – | – | – | – | – |
| > 10 days | ± | – | – | – | – |
| Urease | – | – | – | + | – |
| Growth with: |  |  |  |  |  |
| 10 mg TCH ml^-1^ | ++ | +++ | + | +++ | + |
| 500 mg PNB ml^-1^ | + | ++ | + | ++ | + |
| 5% NaCl | – | – | – | – | – |
| Growth on: |  |  |  |  |  |
| MacConkey agar | – | – | – | – | – |
| Picric acid | – | – | – | – | – |

+++, Strong growth; ++, good growth; +, positive/growth; −, negative/ no growth; ±, variable. ^†^ S, Smooth; W, White; Y, Yellow. ^‡^ N, Non-photochromogenic; S, Scoto-chromogenic
